# Repeated stereotactic body radiotherapy for oligometastatic prostate cancer recurrence

**DOI:** 10.1186/1748-717X-9-135

**Published:** 2014-06-12

**Authors:** Karel Decaestecker, Gert De Meerleer, Bieke Lambert, Louke Delrue, Valérie Fonteyne, Tom Claeys, Filip De Vos, Wouter Huysse, Arne Hautekiet, Gaethan Maes, Piet Ost

**Affiliations:** 1 Department of Urology, Ghent University Hospital, De Pintelaan 185, Ghent, Belgium; 2 Department of Radiotherapy, Ghent University Hospital, De Pintelaan 185, Ghent, Belgium; 3 Department of Nuclear Medicine, Ghent University Hospital, De Pintelaan 185, Ghent, Belgium; 4 Department of Radiology, Ghent University Hospital, De Pintelaan 185, Ghent, Belgium; 5 Department of Radiopharmacy, Ghent University, Harelbekestraat 32, Ghent, Belgium

**Keywords:** Oligometastases, Prostate cancer, Recurrence, Salvage therapy, Stereotactic body radiotherapy

## Abstract

**Purpose:**

To assess the outcome of prostate cancer (PCa) patients diagnosed with oligometastatic disease at recurrence and treated with stereotactic body radiotherapy (SBRT).

**Methods:**

Non-castrate patients with up to 3 synchronous metastases (bone and/or lymph nodes) diagnosed on positron emission tomography - computed tomography, following biochemical recurrence after local curative treatment, were treated with (repeated) SBRT to a dose of 50 Gy in 10 fractions or 30 Gy in 3 fractions. Androgen deprivation therapy-free survival (ADT-FS) defined as the time interval between the first day of SBRT and the initiation of ADT was the primary endpoint. ADT was initiated if more than 3 metastases were detected during follow-up even when patients were still asymptomatic. Secondary endpoints were local control, progression free survival (PFS) and toxicity. Toxicity was scored using the Common Terminology Criteria for Adverse Events.

**Results:**

With a median follow-up from time of SBRT of 2 years, we treated 50 patients with 70 metastatic lesions with a local control rate of 100%. The primary involved metastatic sites were lymph nodes (54%), bone (44%), and viscera (2%). The median PFS was 19 mo (95% CI: 13–25 mo) with 75% of recurring patients having ≤3 metastases. A 2^nd^ and 3^rd^ course of SBRT was delivered in 19 and 6 patients respectively. This results in a median ADT-FS of 25 months (20–30 mo). On univariate analysis, only a short PSA doubling time was a significant predictor for both PFS (HR: 0.90, 95% CI: 0.82 – 0.99) and ADT-FS (HR: 0.83; 95% CI: 0.71 – 0.97). Ten patients (20%) developed toxicity following treatment, which was classified as grade I in 7 and grade II in 3 patients.

**Conclusion:**

Repeated SBRT for oligometastatic prostate cancer postpones palliative androgen deprivation therapy with 2 years without grade III toxicity.

## Background

The standard treatment options for non-castrated prostate cancer (PCa) patients diagnosed with metastatic disease have remained unchanged over the past years [1], with continuous androgen deprivation therapy (ADT) being the cornerstone of treatment [2]. The negative impact of ADT on general health and quality of life has resulted in a search for alternatives [3,4]. Both intermittent ADT and active surveillance are now being considered valuable options in these patients [2].

Like in other solid tumors, there is increasing evidence that patients diagnosed with a limited number of metastases (≤3) – so called “oligometastases” - have a better prognosis compared to patients with extensive metastatic disease [5,6]. This might imply that the oligometastatic status represents a specific metastatic phenotype with a less aggressive behaviour. The clinical implication might be that a localized form of cancer treatment may be effective to delay disease progression [7]. Stereotactic body radiotherapy (SBRT) has proven to be a safe and effective treatment for oligometastases [8].

In the current study we assessed the outcome of PCa patients diagnosed with oligometastatic recurrence and treated with SBRT.

## Methods

This study includes 50 patients diagnosed with ≤ 3 metachronous asymptomatic metastases treated with SBRT at our institution between May 2005 and October 2013. Data for these patients were prospectively collected and analysed. All cases were presented to and approved by the multidisciplinary uro-oncology team and the local ethics committee (EC2011/495). Eligibility criteria included histologically proven diagnosis of PCa and a biochemical relapse following local radical PCa treatment [9]. Exclusion criteria included: serum testosterone level <50 ng/ml at time of detection of metastases, neo-adjuvant or concomitant ADT > 1 month with SBRT or a PSA rise while on active treatment with a luteinizing hormone releasing hormone (LHRH)-(ant)agonist, anti-androgen or estrogens.

All patients were staged with [18 F]-fluorodeoxyglucose (FDG) (n = 32) until 2011, switching to [18 F]-choline positron emitting tomography (PET) until present (n = 18) with co-registered computed tomography (CT) [10]. All scans were interpreted by the radiologist and nuclear medicine physician in consensus reading with knowledge of the clinical history of the patients and of the results of other diagnostic techniques. Every focal tracer accumulation deviating from the physiological distribution of the tracer was regarded as suggestive of disease. A biopsy of the suspected lesions was not routinely performed prior to inclusion and treatment. In case of equivocal findings on PET-CT, an additional magnetic resonance imaging (MRI) of the suspected region was performed (n = 11). Local recurrence was excluded with digital rectal examination in all cases and with multiparametric MRI in patients treated with primary radiotherapy [11,12].

### SBRT technique

All patients underwent a CT-based treatment planning with 2–3 mm slice thickness in supine position with an ankle and knee fix (Sinmed, Cablon Medical, Leiden, The Netherlands). Gross tumor volume (GTV) was delineated using all available clinical, iconographic and metabolic information. A planning target volume (PTV) around the GTV, with margins depending on the site irradiated (2 mm margins for bone metastases, 3 mm for nodes and 5 mm for other sites, except for the liver where a 1 cm margin was used). Organs at risk were delineated, depending on the site of the GTV.

Two radiation schedules were used. For patients treated between 2005 and May 2012, a dose of 50 Gy in 10 fractions of 5 Gy was prescribed to the PTV, combined with a single injection of a short acting (1 month depot) LHRH analogue [13]. For patients treated after this period a median dose of 30 Gy in 3 fractions of 10 Gy was delivered without concomitant LHRH. A switch from 10 fractions to 3 fractions was made for economic and logistic advantages. The normalized total dose of both schedules as calculated with the linear-quadratic model is comparable (87.5 Gy and 90 Gy for 10 × 5 Gy and 3 × 10 Gy, respectively, for an α/β ratio of 2) [14]. Fractions were separated >40 h and <96 h. Treatment was prescribed to the periphery of the PTV (80% of the dose (=30 or 50 Gy), covering 90% of the PTV). The dose was reduced in case of violation of maximal tolerated dose of organs at risks [15]. Intensity modulated radiotherapy with static beams or dynamic arcs was delivered 3 times a week using 6–18 MV photons from a linear accelerator equipped with a multileaf collimator and cone-beam CT (CBCT) (Varian CLINAC, Varian, Palo Alto, CA or Elekta Synergy, Elekta, Crawley, UK).

At each fraction, a CBCT was used for patients’ set-up and target verification, with correction of all shifts without minimal action level. Patients were repositioned in case of detection of rotational errors of non-spherical target volumes exceeding 3 degrees. Automatic matching was done using bone or soft tissue window settings for respectively bone or lymph node metastases. For the patient diagnosed with liver metastasis, a free breathing simulation-CT was fused with PET-CT and MRI. No abdominal compression or fiducials were used. In case of multiple (1 to 3) synchronous lesions, all lesions were treated in the same session and the positioning protocol was repeated per lesion.

### Evaluation of response

The primary endpoint was ADT-free survival (ADT-FS), defined as the time interval between the first day of SBRT and the initiation of palliative ADT. ADT was initiated if more than 3 metastases were detected during follow-up even when patients were still asymptomatic. The type of ADT was left at the discretion of the treating physician. Local progression (LP) was defined as tumor progression within the irradiated PTV. Each metastasis was a target lesion independently assessed for response with the RECIST criteria. In addition, metastases (particularly osseous) with a metabolic complete response on bone or PET scan were scored as complete response in the absence of progression on CT scan. Progression free-survival (PFS) was defined as the absence of new metastases and/or progression of untreated metastases. During treatment, patients were clinically evaluated weekly and at 1 and 3 months thereafter. Follow-up visits with prostate specific antigen (PSA) measurement were scheduled 3-monthly during the first year and 6-monthly thereafter. Reassessments with bone scan and PET/CT imaging was performed in case of 3 rising PSA values after initial response, in case of PSA rise above the pre-SBRT PSA that was confirmed at least once or if clinically indicated to rule out local or distant metastatic progression. In case of an oligometastatic recurrence outside the previous SBRT field, a retreatment with SBRT was offered.

Toxicity was evaluated and graded according to the National Cancer Institute (NCI) Common Terminology Criteria for Adverse Events (CTCAE) v3.0 [16]. Late effects were designated as events occurring > 3 months following treatment or as an event lasting >3 months after treatment.

### Statistics

The Kaplan-Meier method was used to estimate rates of ADT-FS, LP, PFS and prostate cancer specific survival (PCSS). Calculations were done from the start of SBRT. Potential prognostic factors were examined using univariate proportional hazards regression from diagnosis of metastases to start of ADT. Variables exhibiting a p-value ≤ 0.15 in univariate analysis were entered manually in Cox proportional hazards models in a forward stepwise fashion. Variable retention was based on the likelihood ratio test and change in estimated hazard ratios for variables already present. Potential variable selection for univariate analysis was based on previous papers on noncastrate metastases [5,6,16]. The pattern of metastatic spread at time of metastasis was defined as minimal disease in case of involvement of nodes or axial skeleton and as extensive disease as involvement of appendicular skeleton (with or without axial skeleton) or viscera (lung or liver) as suggested previously [16]. Additionally, the total number of metastases was calculated counting all metastatic spots separately. The premetastatic PSA doubling time (PSA DT) was calculated by assuming first-order kinetics and based on all PSA measurements within 1 year (yr) prior to development of noncastrate metastatic disease with a minimum of three measurements, separated by a minimum of 4 weeks. All variables were entered continuously except for risk group at PCa diagnosis: low (T1-T2a and Gleason ≤6 and PSA <10 ng/ml), intermediate (T2b-T2c or Gleason = 7 or PSA 10-20 ng/ml), high (T3a or Gleason 8–10 or PSA > 20 ng/ml) and very high-risk (cT3b-T4 N0 or any T, N1, any Gleason) [9]. Risk groups were based on the final pathological staging in case of surgery. All statistical analyses were performed using SPSS version 21 (SPSS, Chicago, IL) with p <0.05 considered significant.

## Results

### Patient characteristics

Fifty patients were included in the current study. Table 1 summarizes patient and disease characteristics at time of PCa diagnosis and at time of SBRT. The majority of patients was treated with multimodality treatment at PCa diagnosis (Table 1). A pelvic lymph node dissection (PLND) was performed in 32 patients (64%), being positive in 5 patients (10%). During PLND, a median number of 8 nodes was removed. The median interval from PCa diagnosis to first metastatic event was 4.8 yr with a median PSA of 5.1 ng/ml and a PSA DT of 3.8 months (mo) at time of metastases (Table 1). In total 70 metastatic lesions were treated: lymph nodes (54%), bone (44%), and viscera (2%). Thirteen out of 24 patients with a pelvic recurrence had a PLND at initial treatment. The pattern of metastatic spread was categorized as minimal in 70% and extensive in 30% of patients according to the criteria of Yossepowitch et al. [17]. The different subsites of metastatic involvement are depicted in Table 1. The median PSA level at time of detection of metastatic disease was 5.0 ng/ml (range 0.2 – 45.4 ng/ml) compared to 5.1 ng/ml (0.6-116.7 ng/ml) for patients screened with FDG-PET-CT and choline PET-CT, respectively (p = 0.71).

**Table 1:** Patient characteristics

**Characteristics**	**All patients (n = 50)**
**Age at diagnosis (yrs)**	
Median (IQR)	59 (55–62)
**Follow-up from PCa diagnosis (yrs)**	
Median (IQR)	7.8 (5.3-10.5)
**Primary therapy**	
Radical prostatectomy alone	6 (12%)
Radical prostatectomy with postoperative RT	22 (44%)
Radical prostatectomy with postoperative RT + ADT	14 (28%)
Radiotherapy + ADT	6 (12%)
Radiotherapy alone	2 (4%)
**PSA at initial diagnosis (ng/ml)**	
Mean (range)	16 (3.5-81)
Median (IQR)	10.4 (7–16.9)
**Prognostic grouping**	
Low	1 (2%)
Intermediate	16 (32%)
High	19 (38%)
Very high	14 (28%)
**Interval from diagnosis to metastases (yr)**	
Mean (range)	5.3 (0.2 – 15)
Median (IQR)	4.8 (2.9 – 7.3)
**PSA level at first documented metastases (ng/ml)**	
Mean (range)	10.9 (0.2 – 117)
Median (IQR)	5.1 (2.0 – 8.6)
**PSA DT at first documented metastases (mo)**	
Mean (range)	6.0 (1 – 30)
Median (IQR)	3.8 (3.0 – 6.9)
**Number of lesions at diagnosis of metastases**	
1 metastasis	37 (74%)
2 metastases	8 (16%)
3 metastases	6 (12%)
**Primary site of metastases**	
Lymph nodes	
Pelvic	24 (50%)
Obturator	1 (2%)
Internal iliac	6 (12%)
External iliac	10 (20%)
Presacral	2 (4%)
Common iliac	3 (6%)
Combination of nodal sites	2 (4%)
Extrapelvic	1 (2%)
Both	2 (4%)
Bones	
Axial	8 (16%)
Appendicular	11 (22%)
Both	3 (6%)
Viscera	
Liver	1 (2%)
**Treatment at time of metastases (%)**	
SBRT 10 × 5 Gy + 1 mo ADT	35 (70%)
SBRT 3 × 10 Gy	15 (30%)

*Abbreviations*: *yr* year, *mo* months, *IQR* interquartile range.

### Patterns of progression

**Figure 1:**
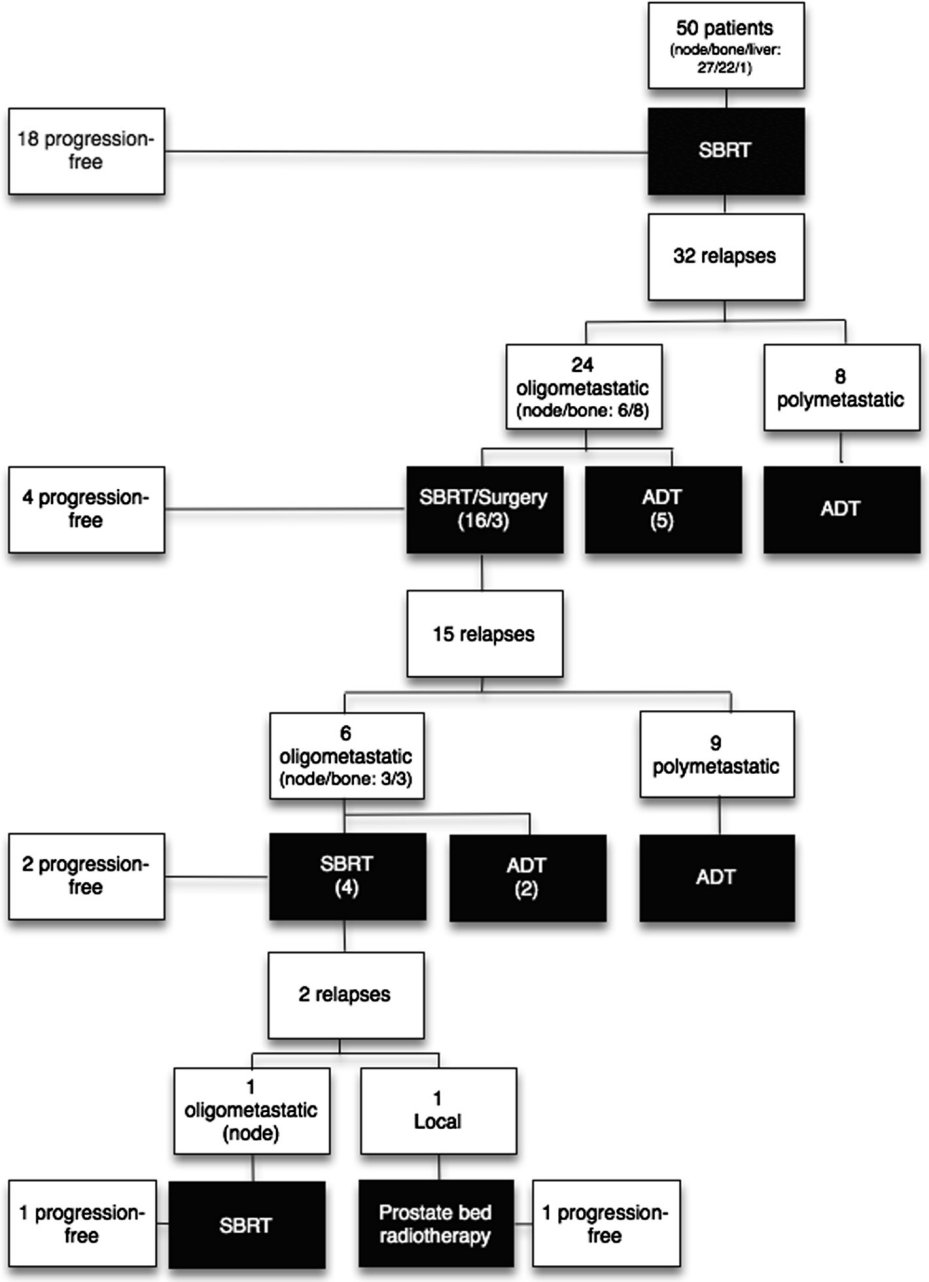
Schematic overview of relapse pattern of oligometastic prostate cancer recurrence following stereotactic body radiotherapy.

**Figure 2:**
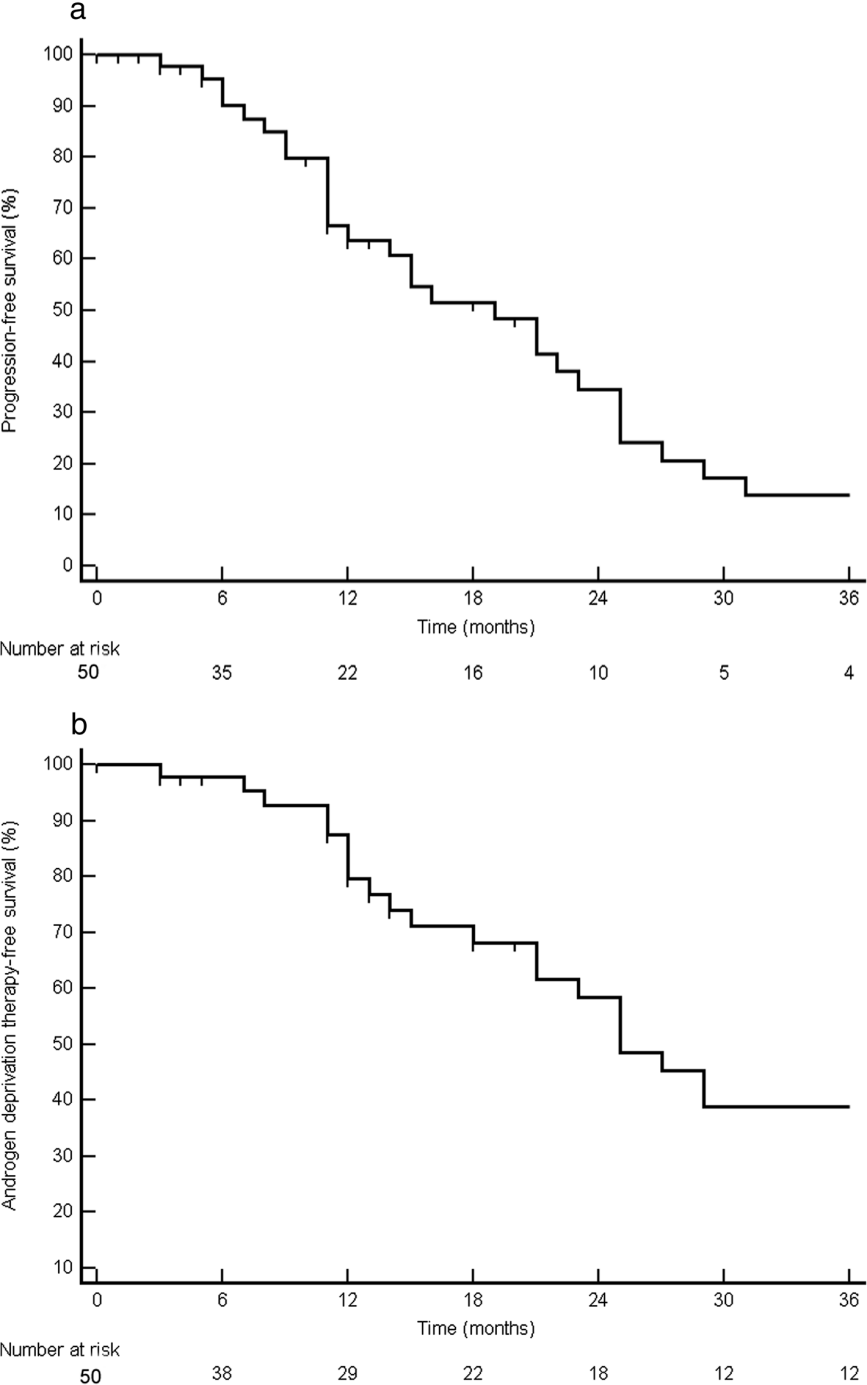
Probability of progression-free survival (a) and androgen deprivation therapy-free survival (b).

**Figure 3:**
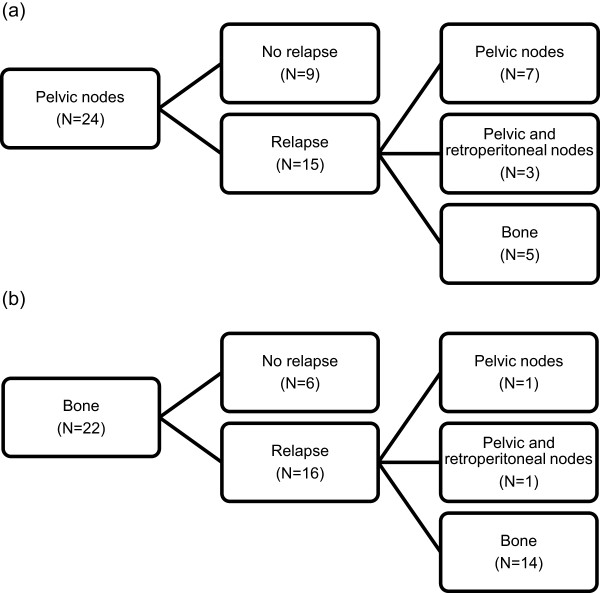
Pattern of relapse of pelvic lymph node (a) and bone metastases (b).

Patterns of first progression following SBRT were recorded and are displayed in Figure 1. After a median follow-up of 2 years (interquartile range, IQR: 8 – 52 mo), 18 patients were disease-free and 32 patients experienced distant metastatic progression, resulting in a median PFS of 19 mo (95% CI: 13–25 mo) (Figure 2a). The 1-year and 2-year PFS rates were 64% and 35% respectively. None of the patients had a local recurrence, resulting in a 100% local control rate. The median PSA at recurrence was 8.5 ng/ml (IQR: 2 – 32 ng/ml) with a median doubling time of 2.7 mo (IQR: 1.5 – 4.7 mo).For patients with initial pelvic lymph node metastases, 67% of the relapses were located in the pelvis or retroperitoneal nodes (Figure 3a) and 33% in the bone. For patients with initial bone metastases, the first site of recurrence following SBRT was located in the bone in 88% of the cases (Figure 3b). Initial progression was again limited to ≤3 metastases in 75% of recurrent patients (N = 24), of which 16 patients received a second course of SBRT and 3 patients received a salvage pelvic lymphadenectomy. The remaining 5 oligometastatic patients and 8 polymetastatic patients received palliative ADT. In the former, the patients refused a second course of SBRT.

At last follow-up, 4 out of 19 patients remain progression-free following a second course of SBRT or salvage surgery. In 6 patients, progression was limited to ≤3 metastases and in 9 patients it exceeded 3 metastases. Four out of 6 oligometastatic patients received a third course of SBRT, while the 2 other patients preferred ADT. The remaining patients received palliative ADT.

One patient relapsed in the prostatic fossa following a 3^rd^ course of SBRT and one patient relapsed with 3 metastatic lesions, while the other 2 remain progression-free at last follow-up. The patient with the local relapse received salvage radiotherapy to the prostate bed and is currently progression-free. The other patient received a 4^th^ course of SBRT to the metastases and is currently progression-free.

**Figure 4:**
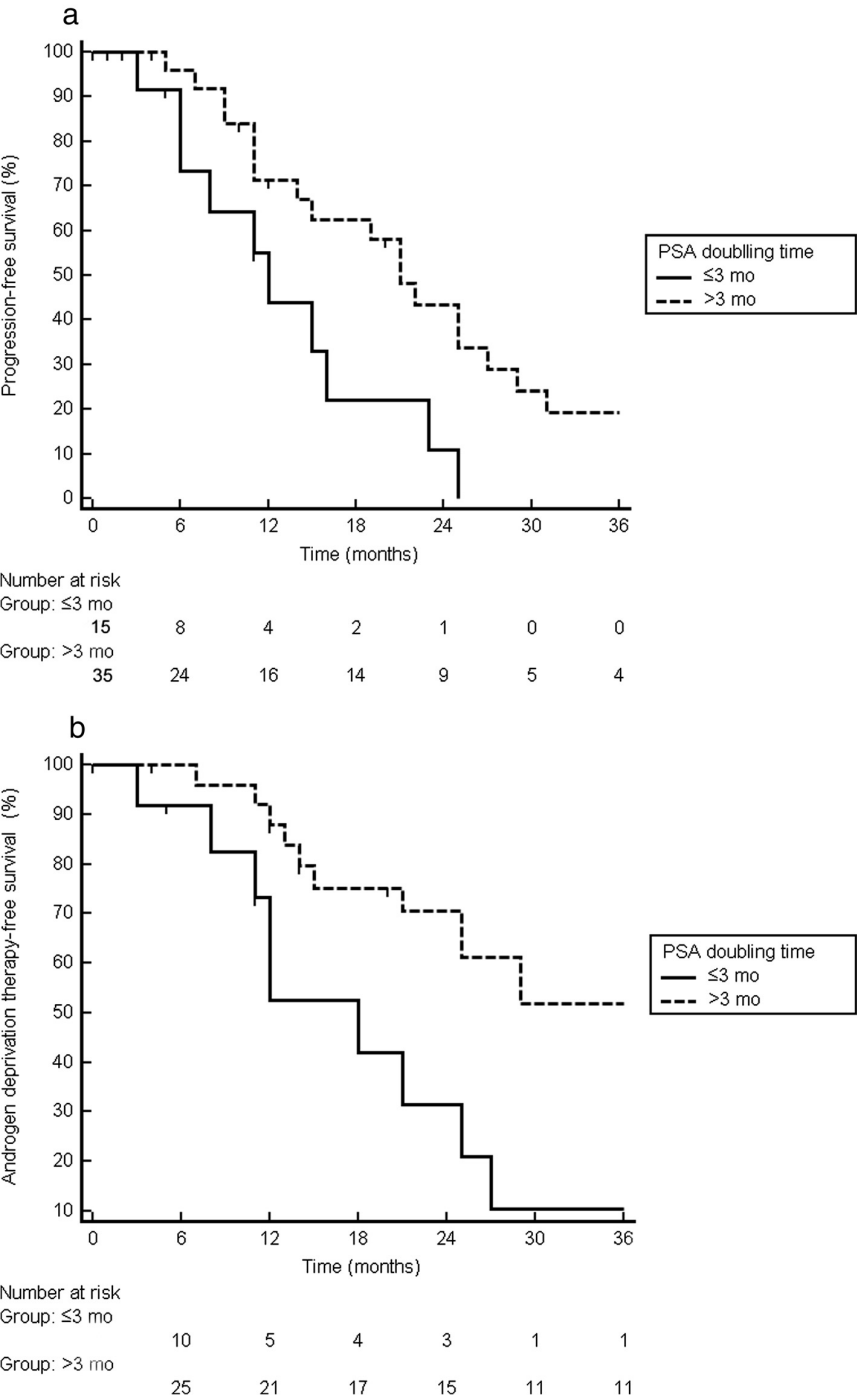
Probability of progression-free survival (a) and androgen deprivation therapy-free survival (b) stratified according to PSA doubling time ≤ 3mo compared to > 3 mo.

To summarize, 26 patients (52%) are progression-free at last follow-up, while 24 patients started with palliative ADT. This results in a median ADT-FS of 25 mo (95% confidence interval, CI,: 20–30 mo) (Figure 2b), with a 1-year and 2-year rate of ADT-FS of 82% and 60% respectively. On univariate analysis, only a short PSA DT prior to SBRT was a significant predictor for both PFS (HR: 0.90, 95%CI: 0.82 – 0.99) and ADT-FS (HR: 0.83; 95%CI: 0.71 – 0.97) (Table 2). The median PFS survival was 12 mo for patients with a DT ≤ 3 mo compared to 21 mo for patients with a longer DT (p = 0.016) (Figure 4a). The median ADT-FS for patients with a PSA DT ≤ 3mo was 18 mo compared to 39 mo for patients with a longer DT (p = 0.014) (Figure 4b). No multivariate analysis was performed as none of the other variables in Table 2 had a p-value ≤ 0.15.

**Table 2:** Univariate Cox proportional hazards model predicting androgen deprivation therapy-free survival and progression-free survival

**Covariate**	**ADT-FS**		**PFS**	
	**HR (95% CI)**	**p-value**	**HR (95% CI)**	**p-value**
Prognostic group at diagnosis				
Low-Intermediate	1	0.72	1	0.41
High	0.99 (0.36 – 2.74)		0.78 (0.33 – 1.88)	0.58
Very high	1.45 (0.48 – 4.4)		1.40 (0.56 – 3.53)	0.47
Interval from diagnosis to metastases (yr)	1 (0.99 – 1.01)	0.51	1 (0.99 – 1.01)	0.55
PSA level at time of metastases (ng/ml)	1 (0.97 – 1.03)	0.96	1 (0.98 – 1.03)	0.67
PSA DT at time of metastases (mo)	0.83 (0.71 – 0.97)	**0.02**	0.90 (0.82 – 0.99)	**0.04**
Number of lesions at diagnosis of metastases	1.11 (0.56 – 2.22)	0.75	1.02 (0.53 – 1.94)	0.96
Pattern of metastatic spread				
Minimal	1	0.37	1	0.27
Extensive	1.48 (0.63 – 3.49)		1.53 (0.72 – 3.2)	
Location of metastasis*				
Node	1	0.10	1	0.25
Bone	2.02 (0.87 – 4.72)		1.54 (0.74 – 3.22)	

*Abbreviation*: *HR* hazard ratio, *CI* confidence interval, *yr* year, *mo* month, *ADT-FS* androgen deprivation therapy-free survival, *PFS* progression-free survival.

*The patient with liver metastasis was excluded from the analysis of this variable. P-values in bold represent significant values <0.05.

Five patients died of prostate cancer, resulting in a 2-year and 5-year PCSS of 96% and 90% respectively. There were no non-prostate cancer related deaths.

### Toxicity

Ten patients (20%) developed toxicity following treatment, which was classified as grade I in 7 and grade II in 3 patients. In case of bone metastasis irradiation, 3 patients reported mild grade I bone pain and 1 patient was diagnosed with an asymptomatic fracture of the ilium on follow-up PET-CT without the necessity of treatment (grade I). In case of SBRT for nodal metastases, 1 patient experienced grade I fatigue and 4 patients experienced diarrhoea (grade I: n = 2 and grade II: n = 2). One patient experienced a worsening of his post-radical prostatectomy urinary incontinence (grade II) six months after SBRT of a pelvic node.

## Discussion

Metastatic prostate cancer is clearly a heterogeneous disease, with the number of metastases at recurrence being recognized as an important prognostic factor [5,6]. However, two major difficulties have complicated assessment of the benefit of radical treatment for oligometastases.

First, the identification of patients with truly oligometastatic disease is inherently challenging. When using bone scintigraphy as a single re-staging modality, the proportion of patients diagnosed with ≤5 lesions is only 41% [18]. With the addition of computed tomography (CT), 73% of patients is diagnosed with ≤3 metastases with a median PSA of 25 ng/ml [5]. With the introduction of more sensitive and specific imaging modalities such as PET-CT and MRI [10,19], oligometastatic disease is detected even earlier at median PSA levels around 7 ng/ml or lower [6,10]. Consequently, both the time between a PSA rise and the detection of metastatic disease is reducing as well as the number of metastases detected [6]. In the current study, the majority of patients were staged with FDG-PET-CT and only a minority with choline PET-CT. Thus, a proportion of patients might have been understaged [10], potentially leading to underestimation of the effect of the treatment. However, the median PSA level at time of detection of metastatic disease was comparable between both FDG and choline in our population. A recent dual-tracer study concluded that although choline appears to be more sensitive than 18 F-FDG for the detection of disease in PSA relapse, 18 F-FDG correlated better with more aggressive disease [20]. Unfortunately, both FDG and choline PET-CT, still underestimate the extent of disease [21]. This is also clearly reflected in our patients treated with SBRT for pelvic lymph nodes, with two out of three patients relapsing in the nodes again. As an alternative to a lesion based approach such as SBRT, the inclusion of an elective nodal volume in the radiotherapy field might reduce these type of relapses. Other groups have started implementing this type of treatment with promising results, however details on the pattern of relapse are lacking [22-24]. Newer tracers, such as (68)Ga-labelled prostate-specific membrane antigen (PSMA), look very promising with a higher yield of lesions with an improved tumor to background ratio [25], but need further validation. Although currently not commercially available, ultrasmall superparamagnetic particles of iron oxide MRI remains one of the most promising imaging modalities for detecting of metastases of normal-sized lymph nodes in PCa [26]. Further improvements in imaging will enable better patient selection for lesion-based therapies.

The second hurdle for delivering radical treatment to metastases by means of radiation was the need for extended fractionation for the delivery of ablative doses to the lesion while avoiding normal tissue toxicity. The development of new radiotherapy planning and treatment technologies have enabled the safe delivery of an equivalent or higher biological effective doses in a reduced number of treatment sessions as compared to a standard 5–7 week course. Nevertheless, SBRT has only been recently implemented for oligometastatic PCa as is demonstrated by the limited number of publications [21,23,27-29]. In a recent systematic review including a mixture of primary solid tumours, it was concluded that SBRT for oligometastatic disease is accompanied with low toxicity and excellent local control [8]. About 20% of patients remain progression free at 2–3 years after SBRT [8]. Our study is in agreement with these findings, showing a 100% local control without grade III toxicity. This supports the 2 fractionation schedules used in our study delivering a biological dose of around 90 Gy. The 2-year progression-free survival of 35% is in line with that of other solid tumors [8]. However, it is on the lower end compared to the other reported PCa series [23,28,29]. This might be partially explained by the fact that most series used concomitant ADT for more than 6 months [23,28,29]. It should also be noted that the number of patients treated is lower in other studies and that most of them only included patients with a single metastasis, which might influence outcome [5,6].

We identified PSA DT as the only variable influencing clinical progression and ADT-FS [5,6]. Initial patient stratification based on PSA DT might help us select the ideal candidates for SBRT. We observed that the pattern of progression after SBRT is most often oligometastatic. Consequently, these patients were offered repeated SBRT to postpone progression to polymetastatic disease requiring systemic treatment. In 50% of the patients we were able to postpone palliative androgen deprivation therapy by at least 2 years. Additionally, this surrogate endpoint describes the proportion of patients who no longer can be ‘salvaged’ with repeat SBRT, as the majority of patients (17 of 24 cases) were started on ADT because of polymetastatic progression. Although promising, the true benefit of SBRT can only be assessed through randomization. A randomized phase II trial comparing active surveillance with eradication of oligometastatic disease by means of SBRT or surgery is halfway recruiting (http://clinicaltrials.gov: NCT01558427). In the meanwhile, SBRT for oligometastatic PCa should be considered investigational.

Despite being the largest series reporting on the outcome of SBRT for oligometastatic PCa, several limitations should be addressed. The median follow-up is too short to reliably report endpoints such as PCSS. The rationale of the addition of single injection of a 1-month preparation of an LHRH-analogue with the initiation of SBRT was to increase radiosensitivity. However, this makes the interpretation of biochemical response increasingly difficult, as all patients had an initial PSA decline. Consequently, we decided to stop the combination therapy from May 2012 onwards to get a clear view on the true benefit of SBRT in this setting. However, the influence ADT on PFS and ADT-FS is probably limited as the duration of testosterone suppression by a 1-month depot of an LHRH- analogue is only between 2 and 4 months depending on the definition of testosterone recovery [30]. It might be hypothesized from the excellent toxicity profile of SBRT that the quality of life in these patients might be superior compared to patients receiving immediate ADT. Unfortunately, these data were not registered in our study, but are currently being prospectively collected.

## Conclusions

Repeated SBRT for oligometastatic prostate cancer postpones palliative androgen deprivation therapy with 2 years without grade III toxicity.

## Competing interests

The authors declare that they have no competing interests.

## Authors’ contributions

KD and PO had full access to all the data in the study and takes responsibility for the integrity of the data and the accuracy of the data analysis. Study concept and design: KD, PO and GDM. Acquisition of data: LD, BL, GM, AH, WH, FDV, VF. Analysis and interpretation of data: KD, PO, GDM. Drafting of the manuscript: KD, PO, GMD, BL. Critical revision of the manuscript for important intellectual content: LD, BL, GDM and VF. Statistical analysis: KD and PO. Supervision: PO. All authors read and approved the final manuscript.
